# Single-agent ibrutinib in RESONATE-2™ and RESONATE™ versus treatments in the real-world PHEDRA databases for patients with chronic lymphocytic leukemia

**DOI:** 10.1007/s00277-019-03830-8

**Published:** 2019-11-19

**Authors:** Gilles Salles, Emmanuel Bachy, Lukas Smolej, Martin Simkovic, Lucile Baseggio, Anna Panovska, Hervé Besson, Nollaig Healy, Jamie Garside, Wafae Iraqi, Joris Diels, Corinna Pick-Lauer, Martin Spacek, Renata Urbanova, Daniel Lysak, Ruben Hermans, Jessica Lundbom, Evelyne Callet-Bauchu, Michael Doubek

**Affiliations:** 1 Centre Hospitalier Lyon-Sud, Hospices Civils de Lyon, Université Claude Bernard, INSERM 1052, Pierre Bénite, France; 2 4th Department of Internal Medicine, Hematology, University Hospital and Faculty of Medicine, Charles University, Hradec Králové, Czech Republic; 3 Department of Internal Medicine, Hematology and Oncology, University Hospital and Faculty of Medicine, Masaryk University, Brno, Czech Republic; 4 ’s-Hertogenbosch, The Netherlands; 5 Janssen Sciences, Dublin, Ireland; 6 Janssen–Cilag Limited, High Wycombe, UK; 7 Janssen Pharmaceuticals, Paris, France; 8 Janssen Pharmaceutica NV, Beerse, Belgium; 9 Janssen-Cilag GmbH, Neuss, Germany; 10 1st Department of Medicine, Department of Hematology, First Faculty of Medicine, Charles University and General University Hospital, Prague, Czech Republic; 11 University Hospital Olomouc, Olomouc, Czech Republic; 12 University Hospital Pilsen, Pilsen, Czech Republic; 13 IQVIA Real-World Insight Solutions, London, UK; 14 CEITEC, Masaryk University, Brno, Czech Republic

**Keywords:** Ibrutinib, Chronic lymphocytic leukemia, Real-world evidence, Randomized controlled trial, Progression-free survival, Overall survival

## Abstract

After analyzing treatment patterns in chronic lymphocytic leukemia (CLL) (objective 1), we investigated the relative effectiveness of ibrutinib versus other commonly used treatments (objective 2) in patients with treatment-naïve and relapsed/refractory CLL, comparing patient-level data from two randomized registration trials with two real-world databases. Hazard ratios (HR) and 95% confidence intervals (CIs) were estimated using a multivariate Cox proportional hazards model, adjusted for differences in baseline characteristics. Rituximab-containing regimens were often prescribed in clinical practice. The most frequently prescribed regimens were fludarabine + cyclophosphamide + rituximab (FCR, 29.3%), bendamustine + rituximab (BR, 17.7%), and other rituximab-containing regimens (22.0%) in the treatment-naïve setting (*n* = 604), other non-FCR/BR rituximab-containing regimens (38.7%) and non-rituximab–containing regimens (28.5%) in the relapsed/refractory setting (*n* = 945). Adjusted HRs (95% CI) for progression-free survival (PFS) and overall survival (OS), respectively, with ibrutinib versus real-world regimens were 0.23 (0.14–0.37; *p* < 0.0001) and 0.40 (0.22–0.76; *p* = 0.0048) in the treatment-naïve setting, and 0.21 (0.16–0.27; *p* < 0.0001) and 0.29 (0.21–0.41; *p* < 0.0001) in the relapsed/refractory setting. When comparing real-world use of ibrutinib (*n* = 53) versus other real-world regimens in relapsed/refractory CLL (objective 3), adjusted HRs (95% CI) were 0.37 (0.22–0.63; *p* = 0.0003) for PFS and 0.53 (0.27–1.03; *p* < 0.0624) for OS. This adjusted analysis, based on nonrandomized patient data, suggests ibrutinib to be more effective than other commonly used regimens for CLL.

## Introduction

Chronic lymphocytic leukemia (CLL) is the most common type of leukemia in adults [1, 2]. CLL primarily affects the elderly, with median age of onset of approximately 70 years [3]. Therapy for CLL has evolved from monotherapy with alkylating agents to chemoimmunotherapy [4, 5], often including an anti-CD20 antibody (e.g., rituximab, ofatumumab, or obinutuzumab) and combinations of fludarabine, cyclophosphamide, bendamustine, or chlorambucil [1, 6–9]. However, conventional CLL therapies, such as fludarabine + cyclophosphamide + rituximab (FCR), may have toxic side effects and are poorly tolerated in frail patients [1, 7]. Therefore, the trade-off between efficacy and toxicity requires consideration of patient fitness, particularly in the elderly [1, 3, 10].

Ibrutinib is a first-in-class, oral, once-daily covalent Bruton’s tyrosine kinase inhibitor approved in the USA [11], Europe [12], and other countries worldwide for patients with treatment-naïve (TN) and relapsed/refractory (R/R) CLL. In randomized registration trials of patients with CLL, ibrutinib significantly improved progression-free survival (PFS) and overall survival (OS) versus chlorambucil in TN patients (RESONATE-2™; NCT01722487) [13] and versus ofatumumab in R/R patients (RESONATE™; NCT01578707) [14]. Additional head-to-head clinical trial comparisons of ibrutinib-based regimens versus other widely used treatments (including current standard-of-care chemoimmunotherapy regimens and other novel regimens incorporated into CLL guidelines [15–17]) are ongoing [18, 19], and recent results from multiple phase 3 trials have been positive [data available as abstracts] [20–22].

Real-world (RW) evidence, derived from the analysis of RW data, is becoming increasingly important in understanding the impact of different diseases and treatments [23, 24]. The Platform for Hematology in Europe, Middle East, Africa (EMEA): Data for Real-life Analyses (PHEDRA) is a unique, noninterventional project based on secondary data collection from RW patient-level databases, and was developed to gain a better insight into the treatment of CLL (and other hematological malignancies) in clinical practice [25]. PHEDRA has three main objectives: (1) to describe treatment patterns; (2) to compare outcomes, PFS and OS, between patients treated with ibrutinib in randomized clinical trials (RCTs) and patients treated with commonly used treatment regimens in clinical practice; (3) to confirm that ibrutinib outcomes in the RCT setting are comparable with outcomes in clinical practice. Methods and findings relating to these objectives in CLL are reported here.

## Subjects and methods

### CLL databases and data extraction

Patient-level data (PLD) for CLL in clinical practice were obtained from independent RW databases from France and the Czech Republic and analyzed retrospectively. The Lyon-Sud dataset provided medical records for patients with CLL diagnosed between 1980 and 2017 at the academic Lyon-Sud Hospital in France, the main treatment center for hematological cancers in the region. The CLLEAR database provided records for CLL patients diagnosed between 1988 and 2017 within the catchment area of contributing local hospitals at seven academic centers in the Czech Republic, located in Brno, Hradec Králové, Nový Jičín, Olomouc, Ostrava, Plzeň, and Prague.

Detailed methods for the PHEDRA project have been published separately [25]. In brief, PLD collected from Lyon-Sud and CLLEAR were transformed into a common data model, which enabled the data to be pooled and analyzed. Data access complied with local data protection rules and regulations. Where required, participation was approved by the local independent ethics committee/institutional review board. PLD were fully anonymized for analyses.

### Treatment patterns

Physicians’ choice (PC) regimens for CLL in clinical practice were identified from the RW databases and analyzed descriptively for the TN and R/R settings (relative to objective 1).

### Adjusted comparisons of RCT and RW data

An adjusted comparison of PFS and OS outcomes was performed on PLD from the ibrutinib arms of RESONATE-2™ (TN CLL) or RESONATE™ (R/R CLL) versus other PC regimens (any treatment regimen being used in clinical practice, except for ibrutinib) in the RW databases (data pooled; objective 2). TN patients were included in the RW cohort for the adjusted comparisons if they met the inclusion criteria of RESONATE-2™ (aged ≥ 65 years and without del17p). All R/R CLL patients from the RW cohort were included in the adjusted comparisons versus RESONATE™.

To account for noncomparability of patient populations due to lack of randomization in RW databases (vs RCTs), a multivariate Cox proportional hazards regression model was used to derive adjusted hazard ratios (HRs) for the relative treatment effect of ibrutinib on PFS and OS versus PC in the RW cohort (i.e., if ibrutinib was used instead of the various actual treatments). Prognostic factors for OS and PFS included in the model were age, sex, and disease stage (based on Binet/Rai) (TN and R/R), as well as line of treatment for the R/R-analysis. The adjusted HRs for treatment effects and prognostic covariates are graphically presented by endpoint using forest plots (point estimate and 95% confidence interval [CI]).

Based on the multivariate model, predicted survival curves for PFS and OS were estimated by patient, using patient-specific baseline characteristics as covariates. A first survival curve represents the predicted patient-specific survival under treatment as observed. A second survival curve represents the predicted survival, simulating the outcomes of these patients as if they were treated with ibrutinib rather than the treatment actually received. The difference between the predicted survival curves represents the adjusted HR for ibrutinib relative to other PC treatments [25].

To assess whether the introduction of novel targeted therapies impacted the results of the adjusted comparisons in the TN or R/R setting, a sensitivity analysis was done, restricting the data to treatments received since 2005 (SA1). As therapies prior and/or subsequent to ibrutinib may have impacted long-term OS outcomes for PC treatments, additional sensitivity analyses were performed on the RW data, excluding all treatment lines following ibrutinib treatment (R/R setting, SA2) and removing all patients treated with ibrutinib from the PC cohort (TN and R/R setting, SA3).

Further sensitivity analyses were conducted in the R/R setting. To evaluate whether the benefit of ibrutinib versus non-ibrutinib PC treatments was consistent across treatment lines, the analyses were done separately for second-line treatment only, and third- and later line treatment.

PFS and OS outcomes in the RW cohort for patients treated with ibrutinib in the R/R setting were compared versus non-ibrutinib–containing PC treatments (objective 3), using a similar methodological approach as described for objective 2. Treatment lines for patients who were initiated on experimental treatment during a later treatment line were censored at the start of the experimental treatment. The number of patients receiving first-line ibrutinib was too low at the time of the data cut to do a similar comparison for TN patients.

### Handling of missing data

To account for missing data on the date of onset of progressive disease in the RW cohort, the date of initiation of the next treatment was assigned as best proxy. As time to the next treatment is expected to be longer than PFS, this approach was considered conservative for ibrutinib [25].

### Unit of observation for RW cohort analyses

The chosen unit of observation for the analyses of RW PLD was the treatment line, rather than the patient [25]: When a patient moved into further treatment lines, second- and subsequent treatment lines were included as separate observations. Patient characteristics captured at the initiation of each treatment line were included as covariates, to reflect their corresponding baseline status at that point.

Using an approach where multiple observations from the same patient are correlated goes against traditional assumptions regarding the independence of observations in statistical analyses. However, this was accounted and controlled for by using the robust sandwich estimate for the covariance matrix [26, 27], which makes standard errors and CIs around point estimates broader than if all observations had come from different patients (and were independent). This is more efficient from a statistical standpoint, as all available information in the data is leveraged in the analyses, provided that appropriate adjustment of the usual variance estimator has been implemented [28].

## Results

**Fig. 1:**
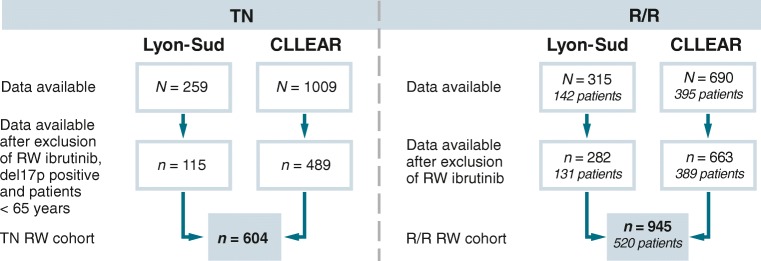
RW database description for Lyon-Sud and CLLEAR*. R/R* relapsed/refractory, *RW* real-world, *TN* treatment-naïve*. N* refers to treatment lines in RW databases. For the TN cohort, the patient number equals the treatment line (i.e., one treatment line per patient). In the R/R cohort, patients could contribute to multiple treatment lines (and both the TN and the R/R analyses)

Table 1 shows baseline and disease characteristics for the RCTs and pooled RW cohort by setting (reported by database in Online Resource Tables 1 [TN] and 2 [R/R]). Figure 1 shows the numbers of patients and treatment lines included in the analysis for the RW cohort.

**Table 1:** Baseline characteristics for TN CLL RW cohort and RESONATE-2™, and for the R/R CLL RW cohort and RESONATE™

	TN		R/R		
	RW cohort (*N* = 604)	RESONATE-2™ ibrutinib arm (*N* = 136)	RW cohort non-ibrutinib (*N* = 945)	RW cohort ibrutinib (*N = 53*)	RESONATE™ ibrutinib arm (*N* = 195)
Median age (range), years	72 (65–96)	73 (65–89)	68 (31–92)	64 (51–81)	67 (30–86)
Age, *n* (%)					
< 60	–	–	193 (20.4)	15 (28.3)	45 (23.1)
60–64	–	–	143 (15.1)	13 (24.5)	32 (16.4)
65–69	201 (33.3)	40 (29.4)	217 (23.0)	11 (20.8)	40 (20.5)
70–74	200 (33.1)	50 (36.8)	169 (17.9)	10 (18.9)	35 (17.9)
75–79	114 (18.9)	24 (17.6)	138 (14.6)	2 (3.8)	29 (14.9)
80+	89 (14.7)	22 (16.2)	85 (9.0)	2 (3.8)	14 (7.2)
Gender, *n* (%)					
Male	370 (61.3)	88 (64.7)	643 (68.0)	35 (66.0)	129 (66.2)
Female	234 (38.7)	48 (35.3)	302 (32.0)	18 (34.0)	66 (33.8)
Binet/Rai stage^a^, *n* (%)					
A/0	82 (13.6)	26 (19.1)	97 (10.3)	10 (18.9)	64 (32.8)
B/I–II	108 (17.9)	63 (46.3)	133 (14.1)	6 (11.3)	30 (15.4)
C/III–IV	178 (29.5)	47 (34.6)	247 (26.1)	8 (15.1)	101 (51.8)
Unknown	236 (39.1)	0 (0.0)	468 (49.5)	29 (54.7)	0 (0.0)
Del17p, *n* (%)					
No	456 (75.5)	134 (98.5)	546 (57.8)	16 (30.2)	132 (67.7)
Yes	–	–	191 (20.2)	18 (34.0)	63 (32.3)
Unknown	148 (24.5)	2 (1.5)	208 (22.0)	19 (35.8)	0 (0.0)
Del11q, *n* (%)					
No	332 (55.0)	107 (78.7)	436 (46.1)	21 (39.6)	132 (67.7)
Yes	134 (22.2)	29 (21.3)	291 (30.8)	9 (17.0)	63 (32.3)
Unknown	138 (22.8)	0 (0.0)	218 (23.1)	23 (43.4)	0 (0.0)
Treatment line, *n* (%)					
Line 2	–	–	495 (52.4)	16 (30.2)	35 (18.0)
Line 3	–	–	235 (24.9)	14 (26.4)	57 (29.2)
Line ≥ 4	–	–	215 (22.7)	23 (43.4)	103 (52.8)
Treatment regimens, *n* (%)					
FCR^b^	177 (29.3)	–	141 (14.9)	–	–
BR	107 (17.7)	–	91 (9.6)	–	–
Chlorambucil	55 (9.1)	–	30 (3.2)	–	–
Anti-CD20 + chlorambucil	59^c^ (9.8)	–	48^d^(5.1)	–	–
Other R	133 (22.0)	–	366 (38.7)	–	–
Other non-R	73 (12.1)	–	269 (28.5)	–	–

*BR* Bendamustine + rituximab, *CLL* Chronic lymphocytic leukemia, *FCR* Fludarabine + cyclophosphamide + rituximab, *R* Rituximab, *R/R* Relapsed/refractory, *RW* Real-world, *TN* Treatment-naïve

*N* refers to patients in RESONATE-2™ and RESONATE™, but refers to treatment lines in RW databases

^a^When Binet stage was missing but Rai stage was available, the Rai stage was assigned as follows: Rai stage 0 = Binet stage A, Rai stages 1–2 = Binet stage B, and Rai stages 3–4 = Binet stage C

^b^FCR may include low-dose regimens (FCR-lite) as well as conventional FCR

^c^Anti-CD20 includes rituximab (*n* = 53) and obinutuzumab (*n* = 6)

^d^Anti-CD20 includes rituximab (*n* = 48)

Other R-containing treatment regimens include FCR-based (TN *n* = 51, R/R *n* = 35), BR-based (TN *n* = 3; R/R *n* = 20), anti-CD20 (TN *n* = 10, R/R *n* = 32), anti-CD20 + chemotherapy (TN *n* = 53, R/R *n* = 235), and other R (not otherwise specified: TN *n* = 16, R/R *n* = 44)

Other (non-R) treatment regimens include alemtuzumab-based (TN *n* = 4, R/R *n* = 111), idelalisib-based (R/R *n* = 26), lenalidomide (R/R *n* = 4), venetoclax (R/R *n* = 6), other chemotherapy (TN *n* = 48, R/R *n* = 88), best supportive care (R/R *n* = 33), and venetoclax combination therapy (TN *n* = 21, R/R *n* = 1)

### Treatment-naïve CLL patients

In the TN setting, including only patients aged ≥ 65 years and without del17p (and excluding ibrutinib treatment, *n* = 5), PLD from 115 and 489 patients in the Lyon-Sud and CLLEAR databases, respectively, were analyzed as the TN RW cohort (pooled number of patients, *n* = 604). Median age was 72 and 73 years, and 61.3% and 64.7% of patients were male, for the RW cohort and RESONATE-2™, respectively. Median follow-up was 30.0 months (Lyon-Sud: 69.0 months; CLLEAR: 23.1 months) and 29.1 months, respectively (Table 1) [13].

#### Description of PC treatments from the RW databases

The most commonly used treatment regimens in TN patients were rituximab-based therapy (*n* = 417 [69.0%]), including FCR (*n* = 177 [29.3%]), bendamustine + rituximab (BR; *n* = 107 [17.7%]), and other rituximab-containing regimens (*n* = 133 [22.0%]), anti-CD20 + chlorambucil (*n* = 59 [9.8%]), and chlorambucil alone (*n* = 55 [9.1%]) (Table 1).

#### Comparison of outcomes with RCT ibrutinib (RESONATE-2™) versus PC treatments from the RW databases

Across all treatments, multivariate analysis of combined data from the RW databases and RESONATE-2™ identified older age as an independent risk factor impacting PFS and OS; male sex was another independent risk factor for OS. There was a strong trend for advanced disease stage to be associated with decreased survival outcomes (Online Resource Fig. 1a, b). These risk factors were included in the Cox proportional hazards model.

**Fig. 2:**
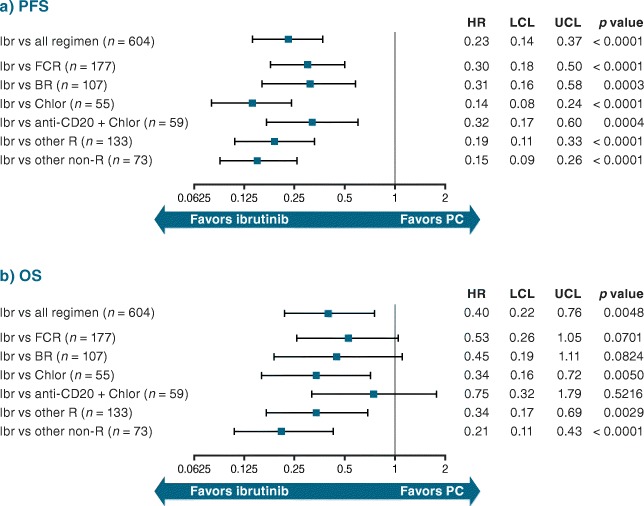
Adjusted HR (95% CI) for (**a**) PFS and (**b**) OS: RESONATE-2™ versus RW TN cohort. Indirect comparison of data from ibrutinib arm of RESONATE-2™ with patient-level data from RW cohort*. BR* bendamustine + rituximab, *Chlor* chlorambucil, *CI* confidence interval, *FCR* fludarabine + cyclophosphamide + rituximab, *HR* hazard ratio, *Ibr* ibrutinib, *LCL* lower confidence limit, *OS* overall survival, *PC* physicians’ choice, *PFS* progression-free survival, *R* rituximab, *RW* real-world, *TN* treatment-naïve, *UCL* upper confidence limit

**Fig. 3:**
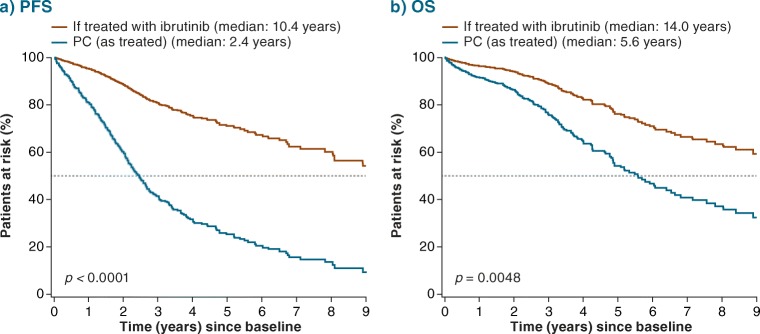
Predicted (**a**) PFS and (**b**) OS: RESONATE-2™ versus RW TN cohort. *p* value for ibrutinib versus PC. Median values are for PFS and OS (rather than follow-up). *OS* overall survival, *PC* physicians’ choice, *PFS* progression-free survival, *RW* real-world, *TN* treatment-naïve

The adjusted HR for ibrutinib versus PC therapy (pooled regimens) was 0.23 for PFS (95% CI 0.14–0.37; *p* < 0.0001) (Fig. 2a) and 0.40 for OS (95% CI 0.22–0.76; *p* = 0.0048) (Fig. 2b), versus the unadjusted HRs of 0.23 (95% CI 0.14–0.37; *p* < 0.0001) and 0.46 (95% CI 0.25–0.85; *p* = 0.0142), respectively (Online Resource Fig. 2a, b). Adjusted HRs for PFS for ibrutinib versus specific regimens (including FCR and BR) ranged between 0.32 (95% CI 0.17–0.60; *p* = 0.0004) for anti-CD20 + chlorambucil and 0.14 (95% CI 0.08–0.24; *p* < 0.0001) for chlorambucil (Fig. 2a). The adjusted HRs for OS ranged between 0.75 (95% CI 0.32–1.79; *p* = 0.5216) for anti-CD20 + chlorambucil and 0.21 (95% CI 0.11–0.43; *p* < 0.0001) for other regimens (see Table 1 footnote for definition) (Fig. 2b). Figure 3a, b shows predicted survival curves (derived from the multivariate Cox model) for OS and PFS, reflecting outcomes for the RW patient cohort as treated versus estimated outcomes for these same patients if they would have been treated with ibrutinib. The difference between the predicted curves reflects the adjusted HRs and provides a visual estimation of improved survival for ibrutinib over other treatments.

Results were similar when only including patients treated since 2005 (*n* = 590 treatment lines, SA1): adjusted HRs for PFS and OS were 0.22 (95% CI 0.14–0.36; *p* < 0.0001) and 0.44 (95% CI 0.24–0.83; *p* = 0.0101), respectively. Removing all patients treated with RW ibrutinib from the PC cohort (*n* = 7, SA3) did not impact the comparisons for PFS and OS (Online Resource Fig. 3a, b).

### Relapsed/refractory CLL patients

After excluding ibrutinib (*n* = 53 treatment lines), PLD were available from 131 patients (282 treatment lines) and 389 patients (663 treatment lines) in Lyon-Sud and CLLEAR, respectively (pooled number of patients, *n* = 520; pooled number of treatment lines analyzed, *n* = 945) (Fig. 1). The ibrutinib arm of RESONATE™ provided data for 195 patients.

In the RW R/R CLL cohort and RESONATE™, the median age was 68 and 67 years, 68.0% and 66.2% of patients were male, and median follow-up was 38.4 (Lyon-Sud: 70.0 months; CLLEAR: 31.7 months) and 44.0 months, respectively [13].

#### Description of PC treatments from the RW databases

The most commonly used PC regimens across all lines for R/R CLL were other rituximab-containing regimens (outside of FCR and BR) (*n* = 366 [38.7%]), other (non-rituximab–containing) regimens (*n* = 269 [28.5%]), FCR (*n* = 141 [14.9%]), BR (*n* = 91 [9.6%]), anti-CD20 + chlorambucil (*n* = 48 [5.1%]), and chlorambucil (*n* = 30 [3.2%]) (Table 1).

#### Comparison of outcomes with ibrutinib (RESONATE™) versus PC treatments from the RW databases

Multivariate analysis using the Cox proportional hazards model of the pooled dataset combining the RW R/R cohort and RESONATE™ data identified older age, advanced disease stage, and later lines of therapy as independent risk factors for both OS and PFS. There was also a clear trend for male sex to be an independent risk factor for both endpoints (Online Resource Fig. 1a, b).

**Fig. 4:**
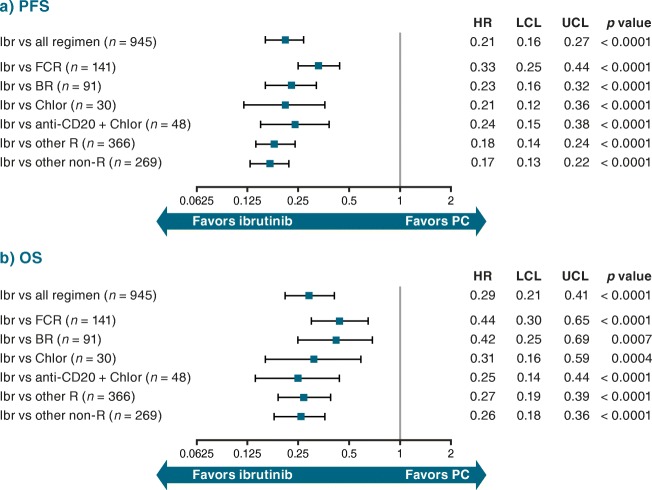
Adjusted HR (95% CI) for (**a**) PFS and (**b**) OS: RESONATE™ versus RW R/R cohort. Indirect comparison of data from ibrutinib arm of RESONATE™ with patient-level data from RW cohort. *BR* bendamustine + rituximab, *Chlor* chlorambucil, *CI* confidence interval, *FCR* fludarabine + cyclophosphamide + rituximab, *HR* hazard ratio, *Ibr* ibrutinib, *LCL* lower confidence limit, *OS* overall survival, *PC* physicians’ choice, *PFS* progression-free survival, *R* rituximab, *R/R* relapsed/refractory, *RW* real-world, *UCL* upper confidence limit

Adjusted HRs for ibrutinib RCT data versus pooled PC treatment regimens from the RW databases were 0.21 (95% CI 0.16–0.27; *p* < 0.0001) for PFS (Fig. 4a) and 0.29 (95% CI 0.21–0.41; *p* < 0.0001) for OS (Fig. 4b) versus unadjusted HRs of 0.28 (95% CI 0.22–0.35; *p* < 0.0001) and 0.43 (95% CI 0.33–0.56; *p* < 0.0001), respectively (Online Resource Fig. 5a, b).

**Fig. 5:**
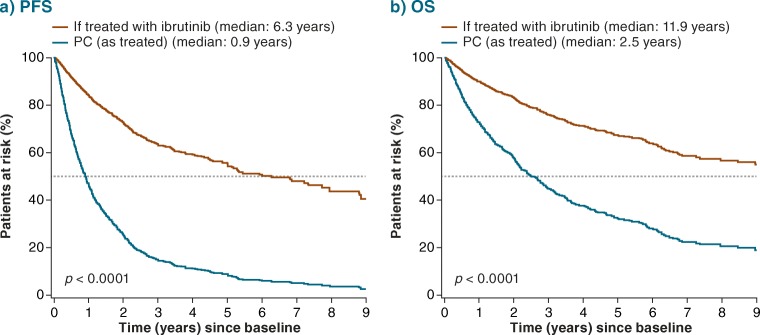
Predicted (**a**) PFS and (**b**) OS for R/R CLL: RESONATE™ versus RW R/R cohort. *p* value for ibrutinib versus PC. Median values are for PFS and OS (rather than follow-up). *CLL* chronic lymphocytic leukemia, *OS* overall survival, *PC* physicians’ choice, *PFS* progression-free survival, *R/R* relapsed/refractory, *RW* real-world

The adjusted HRs for PFS for ibrutinib versus commonly used PC treatment regimens from the RW databases ranged between 0.33 (95% CI 0.25–0.44; *p* < 0.0001 for FCR) and 0.17 (95% CI 0.13–0.22; *p* < 0.0001 for “other” regimens) (Fig. 4a). For OS, the adjusted HRs ranged between 0.44 (95% CI 0.30–0.65; *p* < 0.0001 for FCR) and 0.25 (95% CI 0.14–0.44; *p* < 0.0001 for anti-CD20 + chlorambucil) (Fig. 4b). Figure 5 a and b represent the predicted survival curves for PFS and OS, respectively, in the R/R setting.

The overall R/R results were similar when excluding patients treated before 2005 (*n* = 924 treatment lines, SA1): adjusted HRs for PFS and OS were 0.21 (95% CI 0.16–0.27; *p* < 0.0001) and 0.30 (95% CI 0.21–0.41; *p* < 0.0001), respectively. Additional sensitivity analyses showed that excluding post-ibrutinib treatment lines (SA2) or removing all patients treated with ibrutinib from the RW PC cohort (SA3) did not impact the outcomes (Online Resource Fig. 3a, b).

**Fig. 6:**
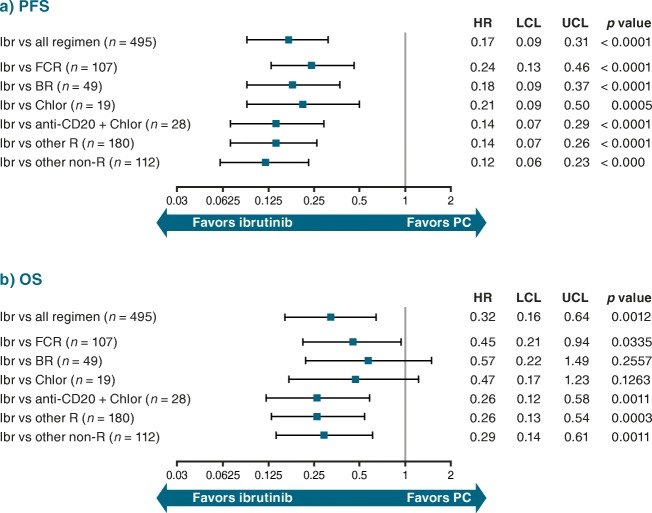
Adjusted HR (95% CI) for (**a**) PFS and (**b**) OS for CLL second-line therapy: RESONATE™ versus RW R/R cohort. Indirect comparison of data from ibrutinib arm of RESONATE™ with patient-level data from RW cohort. *BR* bendamustine + rituximab, *Chlor* chlorambucil, *CI* confidence interval, *CLL* chronic lymphocytic leukemia, *FCR* fludarabine + cyclophosphamide + rituximab, *HR* hazard ratio, *Ibr* ibrutinib, *LCL* lower confidence limit, *OS* overall survival, *PC* physicians’ choice, *PFS* progression-free survival, *R* rituximab, *R/R* relapsed/refractory, *RW* real-world, *UCL* upper confidence limit

**Fig. 7:**
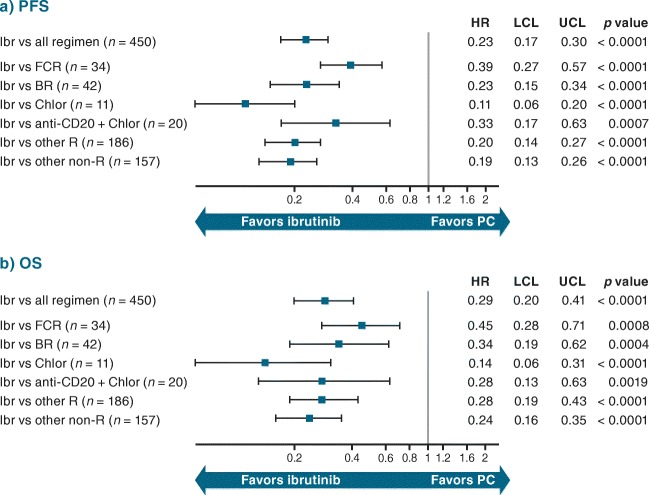
Adjusted HR (95% CI) for (**a**) PFS and (**b**) OS for CLL third- and later line therapy: RESONATE™ versus RW R/R cohort. Indirect comparison of data from ibrutinib arm of RESONATE™ with patient-level data from RW cohort. *BR* bendamustine + rituximab, *Chlor* chlorambucil, *CI* confidence interval, *CLL* chronic lymphocytic leukemia, *FCR* fludarabine + cyclophosphamide + rituximab, *HR* hazard ratio, *Ibr* ibrutinib, *LCL* lower confidence limit, *OS* overall survival, *PC* physicians’ choice, *PFS* progression-free survival, *R* rituximab, *R/R* relapsed/refractory, *RW* real-world, *UCL* upper confidence limit

Adjusted HRs for PFS and OS (including age, sex, and disease stage in the model) for ibrutinib (*n* = 35) versus the PC regimens (*n* = 495) in second-line treatment only from the RW databases were 0.17 (95% CI 0.09–0.31; *p* < 0.0001) and 0.32 (95% CI 0.16–0.64; *p* = 0.0012), respectively (Fig. 6a, b). HRs and 95% CIs for ibrutinib versus individual regimens second-line were similar to those for ibrutinib (*n* = 160) versus the PC regimens (*n* = 450) in third- and later line treatment: 0.23 (95% CI 0.17–0.30; *p* < 0.0001) for PFS and 0.29 (95% CI 0.20–0.41; *p* < 0.0001) for OS (Fig. 7a, b).

#### Comparison of outcomes from the RW databases with ibrutinib versus PC treatments from the RW databases

**Fig. 8:**
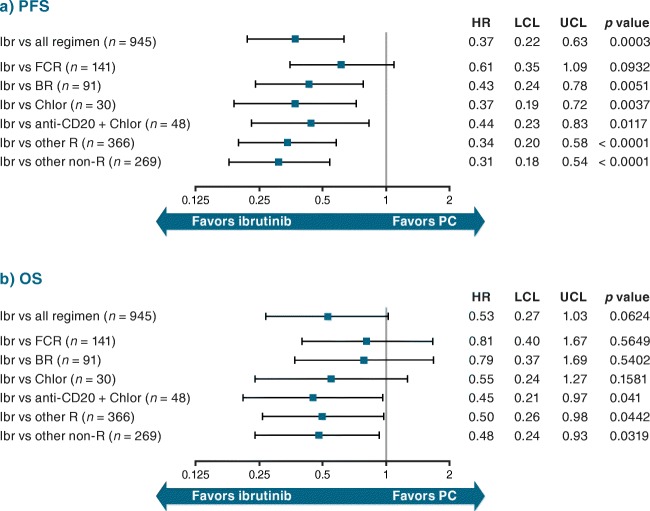
Adjusted HR (95% CI) for (**a**) PFS and (**b**) OS for R/R CLL: ibrutinib RW data versus PC^a^. ^a^PC cohort excludes any ibrutinib RW patients. *BR* bendamustine + rituximab, *Chlor* chlorambucil, *CI* confidence interval, *CLL* chronic lymphocytic leukemia, *FCR* fludarabine + cyclophosphamide + rituximab, *HR* hazard ratio, *Ibr* ibrutinib, *LCL* lower confidence limit, *OS* overall survival, *PC* physicians’ choice, *PFS* progression-free survival, *R* rituximab, *R/R* relapsed/refractory, *RW* real-world, *UCL* upper confidence limit

Adjusted HRs for PFS and OS for ibrutinib (*n* = 53 patients/treatment lines; Table 1) versus PC regimens in the R/R setting (*n* = 945 treatment lines) were 0.37 (95% CI 0.22–0.63; *p* = 0.0003) for PFS (Fig. 8a) and 0.53 (95% CI 0.27–1.03; *p* = 0.0624) for OS (Fig. 8b), versus unadjusted HRs of 0.44 (95% CI 0.26–0.74; *p* = 0.0022) and 0.61 (95% CI 0.31–1.18; *p* = 0.1372), respectively (Online Resource Fig. 6a, b). These results were consistent versus individual PC regimens, except for FCR (0.61 [0.35–1.09]; *p* = 0.0932) for PFS, and FCR (0.81 [0.40–1.67]; *p* = 0.5649) and BR (0.79 [0.37–1.69]; *p* = 0.5402) for OS. The adjusted HR was statistically significant in favor of ibrutinib for OS versus anti-CD20 + chlorambucil, other rituximab-containing regimens, and other regimens (*p* < 0.05; Fig. 8b). The difference between the predicted survival curves (Online Resource Fig. 7a [PFS] and 7b [OS]) for ibrutinib versus PC treatment represents the corresponding adjusted HRs for this comparison.

## Discussion

Registrational phase 3 RCTs demonstrated the significant clinical benefits of ibrutinib in TN and R/R CLL versus chlorambucil (RESONATE-2™) and ofatumumab (RESONATE™), respectively [13, 14, 29, 30]. However, CLL treatment has advanced over the past few years, with an increase in available options for patients with TN or R/R disease. It is therefore informative to consider the comparative effectiveness of ibrutinib with other PC regimens being used in clinical practice.

In the current retrospective analyses, an adjusted comparison was performed using PLD on ibrutinib outcomes from RCTs versus outcomes for PC regimens from two existing RW databases in France and the Czech Republic. In the TN setting, the adjusted HRs for PFS and OS comparing ibrutinib from the RESONATE-2™ study and the RW PC cohort suggest a 4.3-fold improvement in PFS (i.e., the inverse of the reported HR of 0.23) and 2.5-fold (HR = 0.40) improvement in OS with ibrutinib. Comparative results for ibrutinib versus chlorambucil are in line with and confirm available RCT evidence in TN patients with CLL [13]. The adjusted comparison yielded similar findings in the R/R setting, with an estimated 4.8-fold (HR = 0.21) improvement in PFS and 3.4-fold (HR = 0.29) improvement in OS with ibrutinib versus PC regimens from the RW databases; results specific to anti-CD20 therapy are in line with those of the RESONATE™ trial, which had ofatumumab as a comparator [14]. These data show that ibrutinib is consistently associated with improved survival outcomes compared with other PC treatments across lines of therapy.

Sensitivity analyses excluding treatments received prior to 2005 did not impact the results in the TN or R/R setting and illustrate that ibrutinib therapy maintains its benefits compared with newer therapies and novel treatments, including anti-CD20 agents.

Other published analyses support the current findings, showing the clinical benefits of ibrutinib outside of RCTs [31]. Using similar methodology to the current study, PLD were collected from an observational cohort of R/R patients (*n* = 144) diagnosed with CLL between 2002 and 2013 in Sweden, and the ibrutinib arm of RESONATE™. Comparing survival outcomes for ibrutinib versus previous standard-of-care regimens used in second or later lines, the HRs (adjusted for age, sex, disease stage, performance status, and line of therapy) were 0.15 (*p* < 0.0001) for PFS and 0.36 (*p* < 0.0001) for OS, consistent with the results reported in this manuscript. HRs in line with these results have also been reported in studies using a similar modeling approach to compare PLD for single-agent ibrutinib from RESONATE™ with BR from the HELIOS trial (0.13 for PFS and 0.45 for OS) [data available as an abstract] [32].

The benefit of ibrutinib in the RW has also been shown to be comparable with that observed in clinical trials when analyzing data from patient access programs. Analysis of a series of 372 efficacy-evaluable patients with poor prognosis, R/R CLL from the French Temporary Authorization for Use database (December 2013 to November 2014) reported a best overall response rate of 88.5% with ibrutinib treatment after a median follow-up of 3 months [33] (similar to the 90% response rate reported in RESONATE™ at 12 months [14]). Results for 95 poor prognosis patients with CLL who were treated with ibrutinib in a Swedish compassionate use program (from May 2014 to May 2015) found in patients with R/R disease, with median follow-up of 10.2 months, the overall response rate was 84%, and 77% of patients were progression free (vs 88% at 6 months in RESONATE™) [14, 34]. A study of 216 R/R patients with CLL included in the named patient program for ibrutinib in Italy (from April 2014 to January 2015) reported an 80% response rate and PFS rate of 65% at 24 months [data available as an abstract] [35]. In addition, an observational retrospective study of data from 2908 patients with R/R CLL enrolled in the international named patient program for ibrutinib (from March 2014 to March 2015) found that estimated time on treatment (which broadly reflects PFS) in the RW was also similar to that observed in RESONATE™. Multivariate analysis showed that younger age (< 50 years) and achievement of complete or partial response to prior therapy were independent factors significantly associated with longer time on treatment [36].

It is important to note that, since our comparative analyses were conducted, additional data have become available from multiple phase 3 RCTs investigating ibrutinib in comparison with more modern and aggressive chemoimmunotherapy regimens for TN CLL than utilized in RESONATE-2™. These recent trial results support the benefit of ibrutinib-based regimens compared with modern standard therapeutic options. In the E1912 trial of young (≤ 70 years of age), fit patients with TN CLL, ibrutinib combined with rituximab led to superior PFS and OS compared with FCR [20], whereas in the ALLIANCE A014202 trial of fit, older patients (≥ 65 years of age), first-line treatment with single-agent ibrutinib or ibrutinib + rituximab significantly prolonged PFS compared with BR [21]. Further, in the iLLUMINATE study of patients with CLL and ≥ 65 years of age (or younger but with comorbidities), a chemotherapy-free regimen of ibrutinib + obinutuzumab improved PFS, response rates, and depth of remission compared with chlorambucil + obinutuzumab chemoimmunotherapy, irrespective of the presence of high-risk genomic features [22]. These RCT findings are likely to be practice changing in the TN setting. A recent cross-comparison of RCT data for ibrutinib from RESONATE-2™ and chemoimmunotherapy regimens from CLL8 (FCR), CLL10 (FCR and BR), CLL11 (obinutuzumab + chlorambucil and rituximab + chlorambucil), and COMPLEMENT-1 (ofatumumab + chlorambucil) found that PFS appeared longer with ibrutinib and OS was similar; in trials excluding patients with del17p (CLL10), or including older or less fit patients (CLL11), ibrutinib prolonged PFS in high-risk subgroups [37]. Although firm conclusions cannot be drawn from this analysis, the results are generally supportive of the favorable outcomes provided by ibrutinib in TN CLL populations compared with alternative standard regimens.

In the R/R setting, the current PHEDRA study is the first to compare outcomes for ibrutinib with other PC treatments in CLL patients from RW databases. HRs for PFS and OS confirmed the results of the ibrutinib RCT and RW database PC treatment comparison, suggesting that ibrutinib is more effective than several other frequently used regimens. However, results suggest the relative benefit (expressed as an HR) to be less in favor of ibrutinib than the RCT-based ibrutinib versus PC treatment comparison. As ibrutinib was first evaluated and approved for a more difficult to treat CLL population (i.e., in R/R disease/later lines of treatment, patients with del17p [14]), physicians might tend to reserve its use in patients with more advanced disease, or high-risk cytogenetic abnormalities, in clinical practice. Such channeling bias could then have led to a conservative estimate of the clinical benefit of ibrutinib versus PC treatment. There is also some uncertainty around the findings of the comparison of RW data owing to the small sample size for RW ibrutinib (*n* = 53), as reflected in the wide CIs. Further comparisons of outcomes with RW ibrutinib data versus PC treatment in both the TN and R/R CLL settings will be conducted as further data become available.

Our results should be interpreted in light of the following potential limitations. Firstly, estimates on OS benefit when comparing ibrutinib with specific regimens often had large CIs, due to the low number of events and small patient numbers. Pooling data from both RW databases helped to increase sample sizes and statistical power. Secondly, despite all attempts to adjust for differences between both compared patient populations, residual confounding cannot be excluded, as is the case for any nonrandomized comparison.

Some clinical factors, not consistently captured across the different data sources, may have also differed between the populations and may have led to residual bias. For instance, due to the high rate of missing cytogenetic baseline data (i.e., *IGHV*, del11q, or del17p status), mutational status could not be included or adjusted for in the multivariate analysis. However, compared with unadjusted analyses for both PFS and OS, adjustment for any of the available patient characteristics improved the HR in favor of ibrutinib, suggesting that any residual confounding factor may still bias results against ibrutinib. Exploratory analyses comparing the PFS and OS results with the RW database cohort excluding patients with missing del17p status showed only small numerical differences, indicating that inclusion or exclusion of these patients would not change the results.

Finally, the use of time to next treatment as proxy for missing progression dates (in the RW cohort only) may have biased results for PFS against ibrutinib, as time to next treatment is expected to be longer than PFS.

In conclusion, as therapeutic options continue to expand in CLL and there is no single standard-of-care regimen for all patients in the TN or R/R settings, it is worthwhile to compare the outcomes of different treatments in clinical practice as well as in RCTs. Our comparisons of outcomes for ibrutinib and PC treatments from two large high-quality data sources in CLL confirm RCT results, showing improved PFS and OS for ibrutinib over alternative regimens in both the TN and R/R settings. Outcomes for ibrutinib treatment based on RW data were also favorable. These findings extend available evidence on the benefit of ibrutinib versus a range of regimens, including those not yet evaluated in RCTs. Additionally, the results provide information on patient characteristics and treatment patterns, which is important given the absence of a pan-European registry of patients with CLL. Overall, the results from the PHEDRA project can help inform the choice of regimen for CLL across different lines of treatment.
